# Tadalafil ameliorates bladder overactivity by restoring insulin-activated detrusor relaxation via the bladder mucosal IRS/PI3K/AKT/eNOS pathway in fructose-fed rats

**DOI:** 10.1038/s41598-021-87505-3

**Published:** 2021-04-15

**Authors:** Wei-Chia Lee, Steve Leu, Kay L. H. Wu, You-Lin Tain, Yao-Chi Chuang, Julie Y. H. Chan

**Affiliations:** 1 Division of Urology, Kaohsiung Chang Gung Memorial Hospital, and Chang Gung University College of Medicine, Kaohsiung, Taiwan; 2 Institute for Translational Research in Biomedicine, Kaohsiung Chang Gung Memorial Hospital, 123 Ta Pei Rd., Niao Song Qu, Kaohsiung, Taiwan, Republic of China; 3 Department of Pediatrics, Kaohsiung Chang Gung Memorial Hospital, and Chang Gung University College of Medicine, Kaohsiung, Taiwan

**Keywords:** Physiology, Urology

## Abstract

The pathophysiologies of metabolic syndrome (MS) and overactive bladder (OAB) might overlap. Using fructose-fed rats (FFRs) as a rodent model of MS we investigated the effects of tadalafil (a phosphodiesterase type 5 inhibitor) on the dysregulated insulin signalling in the bladder mucosa and bladder overactivity. Micturition behaviour was evaluated. Concentration–response curves on detrusor relaxation to insulin stimulation were examined. Expression and phosphorylation of proteins in the insulin signalling pathway were evaluated by Western blotting. Levels of detrusor cGMP and urinary nitrite and nitrate (NOx) were measured. We observed FFRs exhibited metabolic traits of MS, bladder overactivity, and impaired insulin-activated detrusor relaxation in organ bath study. A high-fructose diet also impeded insulin signalling, reflected by overexpression of IRS1/pIRS1^Ser307^ and pIRS2^Ser731^ and downregulation of PI3K/pPI3K^Tyr508^, AKT/pAKT^Ser473^, and eNOS/peNOS^Ser1177^ in the bladder mucosa, alongside decreased urinary NOx and detrusor cGMP levels. Tadalafil treatment restored the reduced level of mucosal peNOS, urinary NOx, and detrusor cGMP, improved the insulin-activated detrusor relaxation, and ameliorated bladder overactivity in FFRs. These results suggest tadalafil may ameliorate MS-associated bladder overactivity by restoring insulin-activated detrusor relaxation via molecular mechanisms that are associated with preservation of IR/IRS/PI3K/AKT/eNOS pathway in the bladder mucosa and cGMP production in the bladder detrusor.

## Introduction

Overactive bladder (OAB) is a complex of symptoms including urinary urgency, frequency, and nocturia with or without urge incontinence^1^. Metabolic syndrome (MS), a prediabetic condition, is a clustering of cardiovascular risk factors including hypertension, dyslipidaemia, central obesity, and impaired glucose tolerance^2^. Both OAB and MS have high global prevalence (i.e., 16–23% and > 25%, respectively) and exert numerous adverse effects on public health^1–4^. Accumulated evidence suggests that the pathophysiologies of MS and OAB might overlap, and MS may contribute to mechanisms underlying the emergence of OAB^5^.

Dysregulation of urothelial signalling and release of mediators as well as instability of contractile control are key factors in the aetiology of OAB^5^. Evidence from animal studies suggest that metabolic traits of MS may occur with bladder overactivity resultant from urothelial dysfunction^6,7^ and poor detrusor relaxation^7,8^, which mainly involve the endothelial nitric oxide synthase (eNOS)/nitric oxide (NO) system^6,7,9^. Furthermore, the mucosal phosphoinositide 3-kinase (PI3K)/protein kinase B (AKT)/eNOS pathway is known to be engaged in the insulin-induced relaxation of human and mouse bladder^7^. Insulin resistance is a key component of MS. As such, reduced insulin action in the bladder mucosa may play a pivotal role in the manifestation of OAB symptoms under the condition of MS; albeit details in molecular signalling in the MS-associated OAB are not fully delineated.

Tadalafil, a phosphodiesterase type 5 (PDE5) inhibitor, can amplify the NO/cyclic guanosine monophosphate (cGMP) pathway and cause nonvascular smooth muscle relaxation^10^. In 2011, the Food and Drug Administration in the US proved the use of tadalafil for treating patients with lower urinary tract symptoms secondary to benign prostatic hyperplasia. Furthermore, clinical evidence indicates therapeutic potential of tadalafil in OAB patients^11^. Recently, tadalafil was proved to be effective as a treatment of OAB in a phase 3 placebo-controlled trial^12^. Given that MS-associated bladder overactivity is one of the prominent OAB phenotypes, tadalafil might have the potential to become a noteworthy personalized treatment for MS patients^13^. However, the mechanism underlying the beneficial effect of tadalafil on OAB is not fully understood.

Epidemiological observations have shown that fructose consumption is associated with the increasing prevalence of MS^14^; accordingly, the fructose-fed rat (FFR) model has been used as an animal model of MS in the literature^15^. The high fructose diet leads to metabolic dysfunctions, including insulin resistance, hypertension, dyslipidaemia, and bladder overactivity^6,8,15–17^. that resemble metabolic traits of human MS. In the present study, we hypothesized that tadalafil may improve MS-associated bladder overactivity in FFRs through its action to restore the insulin-activated detrusor relaxation via molecular process that involves the IR/IRS/PI3K/AKT/eNOS pathway in the bladder mucosa.

## Results

### General characteristics and micturition behaviour

**Figure 1:**
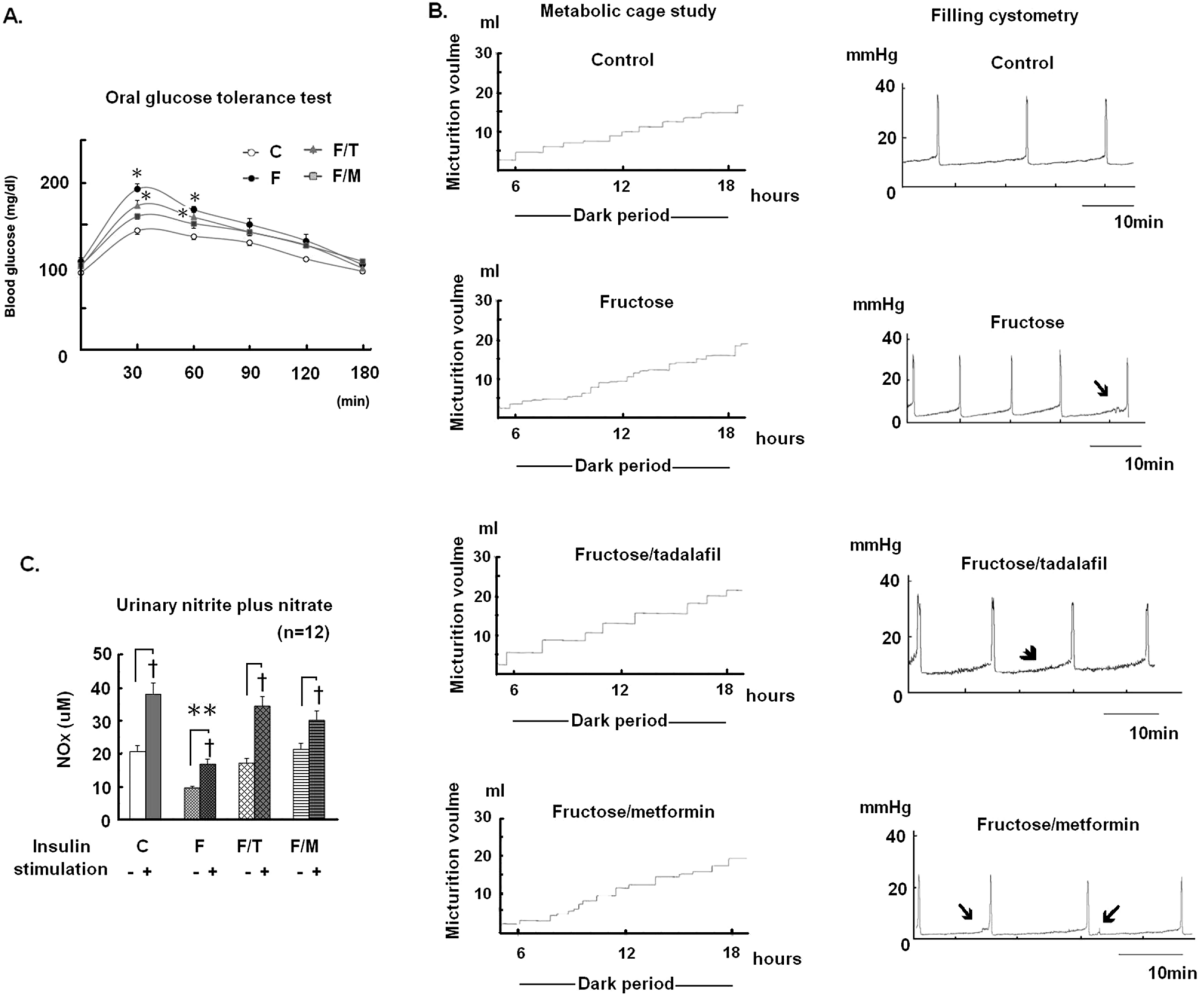
Glucose tolerance, urinary NO content and micturition behavior of experimental animals. (**A**) The response curves of plasma glucose to oral glucose tolerance (OGTT) test, (**B**) metabolic cage study and filling cystometry, and (**C**) levels of urinary nitrite and nitrate (NOx) before and at 2 h after insulin stimulation in control (C) and fructose fed rats (F), alone or with addition of tadalafil (T) or metformin (M) treatment. Data are represented in mean ± SEM, n = 12 animals in each group. *p < 0.05 in comparison with the control group by one-way ANOVA with Dunnett’s test, and ^✝^p < 0.05 in paired *t*-test. Arrows indicate the non-voided contraction, and arrowhead denotes the increased basal tone in (**B**).

Table 1 and Fig. 1 present the general characteristics, micturition behaviour, and biochemical results of all experimental groups (n = 12). No adverse event was found through this experiment. Compared to the control group, the fructose group showed higher mean values in MS traits, including oral glucose tolerance test (OGTT) glucose level (Fig. 1A), mean arterial pressure, serum levels of triglycerides, cholesterol, uric acid, and insulin. The FFRs also exhibited the phenotypes of bladder overactivity, reflected by the increased micturition frequency in the metabolic cage study and a shortened intercontractile interval, the non-voided contraction, and the increased basal tone in cystometry (Fig. 1B). The increased micturition frequency was notably alleviated in FFRs treated with tadalafil. The same treatment, on the other hand, had no discernible effect on MS traits in the same animals. Metformin, a biguanide anti-diabetic medication used in clinical practice, was included in the current study as a control treatment for MS. Metformin treatment significantly reduced hyperinsulinemia and improved OGTT values, as well as bladder overactivity in FFRs. Figure 1C shows the results of urinary nitrite and nitrate (NOx) levels before and after insulin challenge in the experimental animals. In contrast to the pre-test results, all groups had higher post-test urinary NOx levels. Additionally, the fructose group showed significantly lower urinary NOx levels than the controls both before and after insulin challenges. Tadalafil and metformin treatments to the FFRs restored the urinary NOx levels, both before and after insulin challenges, to values comparable to that in the control animals.

**Table 1:** General characteristics of experimental animals.

	Mean ± SEM			
	C	F	F/T	F/M
**General characteristics**				
Body weight (g)	284.5 ± 2.8	289.5 ± 2.7	283.2 ± 5.1	282.2 ± 2.9
Bladder weight (mg)	102.5 ± 2.6	104.5 ± 2.5	104.1 ± 3	105.1 ± 3.9
Mean arterial pressure (mmHg)	123.1 ± 3.8	150.7 ± 3.5*	144.3 ± 2.9*	145.4 ± 2.6*
**Fasting biochemistry parameters**				
Triglycerides (mg/dl)	39.4 ± 1.5	72.4 ± 3.98*	54.4 ± 4.69*	61.1 ± 4.1*
Cholesterol (mg/dl)	61.1 ± 2.6	82.6 ± 1.1*	73.9 ± 2.8*	79.1 ± 1.85*
Creatinine (mg/dl)	0.61 ± 0.01	0.63 ± 0.03	0.69 ± 0.05	0.57 ± 0.01
Uric acid (mg/dl)	1.4 ± 0.04	1.77 ± 0.06*	1.65 ± 0.07*	1.69 ± 0.05*
Glucose (mM)	6.9 ± 0.18	7.5 ± 0.2	7.4 ± 0.19	7.1 ± 0.36
Insulin (mU/l)	11.9 ± 0.6	22.7 ± 1.2*	20.1 ± 1.3*	14.8 ± 0.9
HOMA-IR	3.6 ± 0.2	7.6 ± 0.5*	6.7 ± 0.5*	4.7 ± 0.3*
**Metabolic cage study/24 h**				
Water intake (ml)	35.7 ± 1.7	34.1 ± 1.4	34.5 ± 2.7	32.1 ± 1.9
Urine output (ml)	25.9 ± 1.7	23.5 ± 1.6	25.6 ± 0.9	22.6 ± 1.7
No. voids	19.1 ± 0.7	23.8 ± 1.1*	18.8 ± 0.7	19.5 ± 1.3
**Cystometric parameters**				
Voiding pressure (mmHg)	26.1 ± 1.3	24.6 ± 1.1	25.2 ± 1.2	25.7 ± 1.1
Inter-contractile interval (min)	12.8 ± 0.81	8.8 ± 0.59*	12.7 ± 0.85	11.1 ± 0.96

Data are presented in mean ± SEM, n = 12 in each group. C, control group; F, fructose fed group; F/T. fructose fed animals with addition of datalafil treatment; F/M fructose fed animals with addition of metformin treatment.

### Detrusor relaxation response to insulin

**Figure 2:**
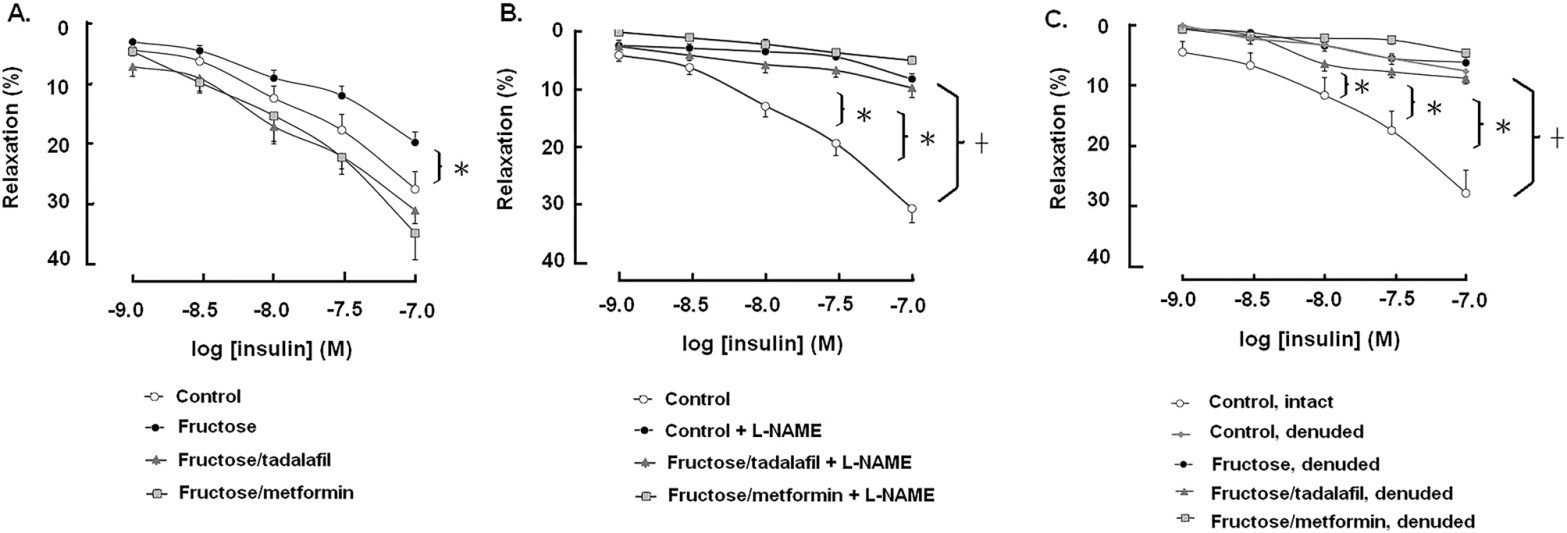
The response curves of relaxation of detrusor muscle in response to insulin (1–100 nM) stimulation in mucosa-intact (n = 8) (**A**), L-NAME-treated (n = 8) (**B**) and mucosa-denuded (n = 6) (**C**) bladder strips of control or fructose fed animals, alone or with addition of L-NAME, tadalafil or metformin. Data are expressed as mean ± SEM. *p < 0.05 in comparison with the control group by one-way ANOVA with Dunnett’s test, and ^✝^p < 0.05 in paired *t*-test. In all experiments, the bladder strips were pre-contracted by the incubation of KCl (80 mM). The means of KCl-induced contractility in the control group are 1.118 g/mm^2^ (**A**), 1.194 g/mm^2^ (**B**), and 1.095 g/mm^2^ (**C**), respectively.

Insulin relaxes urinary bladder through the release of NO from the mucosa layer^7^. We extend the conclusion to study the effect of tadalafil on detrusor relaxation in response to insulin challenge in the control and fructose groups. As shown in Fig. 2A, insulin stimulation produced relaxation of KCl precontracted mucosa-intact bladder strips. Compared with the control group, the fructose group showed a significant impairment in insulin-stimulated detrusor relaxation, which was restored by the treatment with tadalafil or metformin. Inhibition of NOS by the treatment with L-NAME also inhibited the insulin-promoted muscle relaxation, which, at the same time, blocked the protective effects of tadalafil and metformin on the impaired insulin-stimulated relaxation in FFRs (Fig. 2B). Moreover, insulin-stimulated detrusor relaxation observed in the mucosa-intact bladder strips was lessened in the mucosa-denuded detrusor of the control group (Fig. 2C). Interestingly, protection by both tadalafil and metformin on the defected detrusor relaxation in response to insulin in mucosa-intact bladder strips was dispelled in the mucosa-denuded bladder strips of FFRs.

### Assessment of mucosal proteins in the IR/IRS/PI3K/AKT/eNOS pathway, detrusor PDE5 expression and activity, and detrusor cGMP levels

**Figure 3:**
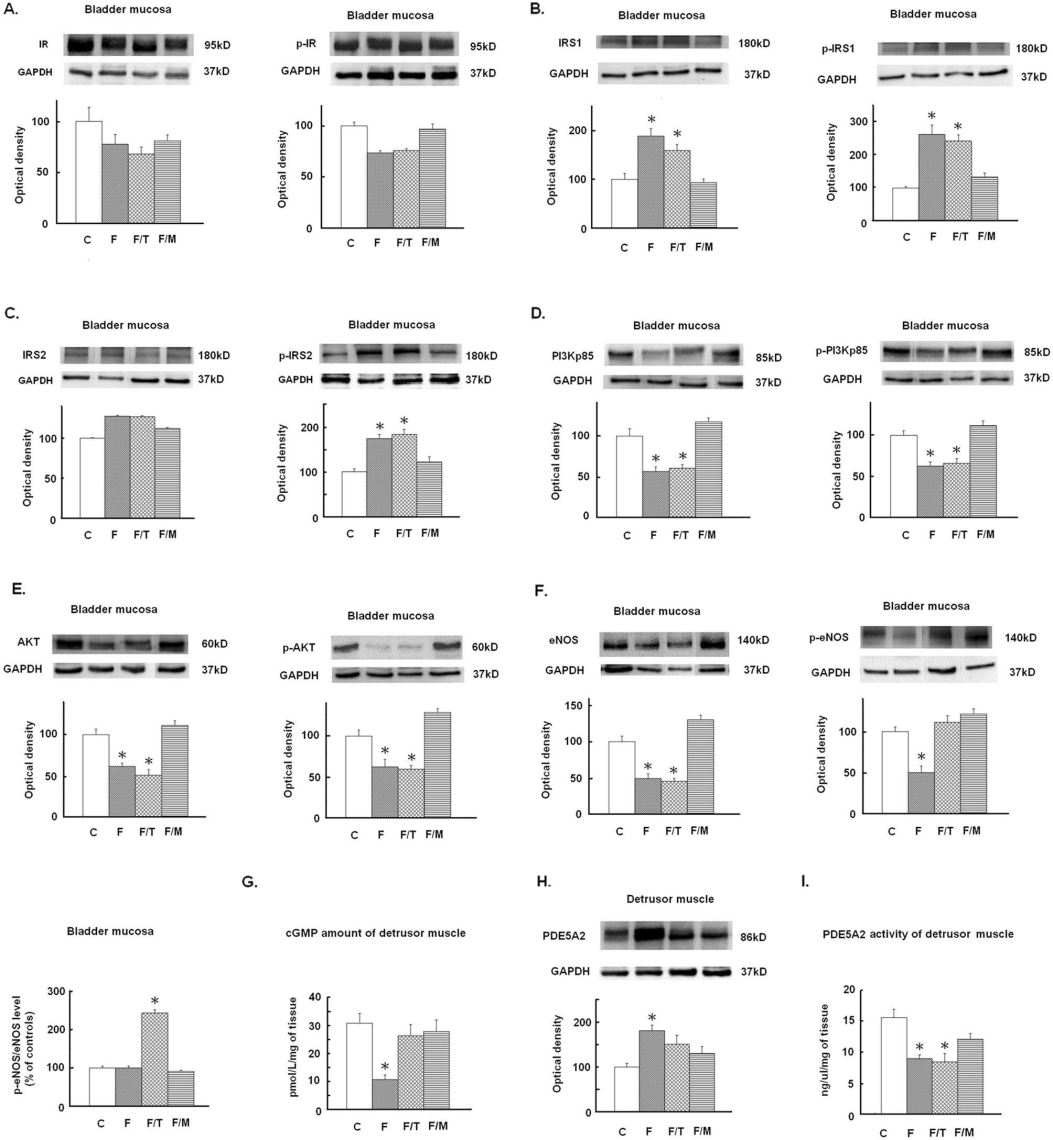
Insulin signalling of IR/IRS/PI3K/AKT/eNOS pathway in mucosa, and cGMP level and PDE5 expression and activity in detrusor in control (C) and fructose fed rats (F), alone or with addition of tadalafil (T) or metformin (M) treatment. Representative Western blot gels and densitometric analysis of total and phosphorylated (p) IR (**A**), IRS1 (**B**), IRS2 (**C**), PI3Kp85 (**D**), Akt (**E**), eNOS (**F**) in the bladder mucosa, as well as cGMP level (**G**) and protein expression (**H**) and activity of PDE5A2 (**I**) in bladder detrusor. The cGMP level and PDE5 activity in detrusor were evaluated by ELISA. Group data are expressed in percentage to the control group denoted as 100%. Data are presented in mean ± SEM, n = 8 in each group. *p < 0.05 in comparison with the control group by one-way ANOVA with Dunnett’s test.

The activation of IR/IRS and subsequent activation of the PI3K/AKT/eNOS signalling mediate detrusor relaxation to insulin stimulation^7,18^. Figure 3 shows protein expressions of the canonical signalling pathway of insulin, which activates IR/IRS/PI3K/AKT/eNOS signalling and promotes NO release in the bladder mucosa to relax the detrusor muscle by stimulating cGMP production. Compared with the control group, the fructose group showed a significant increase in the total content of IRS1 and IRS serine phosphorylation at Ser^307^ for IRS1 and at Ser^731^ for IRS2 (Fig. 3B,C), but a significant decrease in the expression and phosphorylation of downstream effectors of PI3K, AKT, and eNOS (Fig. 3D–F); all of which, except the phosphorylation at Ser^1175^ of eNOS (Fig. 3F), were not significantly affected by the tadalafil treatment. In contrast, metformin treatment notably restored the expression of these proteins to levels not different to the control group. Furthermore, fructose group, but not the tadalafil- or metformin-treated FFRs, exhibited a lower cGMP level than the control group in the detrusor muscle (Fig. 3G). The fructose group also presented a higher PDE5A2 expression in the detrusor than controls (Fig. 3H); while both the fructose and the fructose plus tadalafil-treated group had a lower PDE5A2 activity than the control group (Fig. 3I).

## Discussion

**Figure 4:**
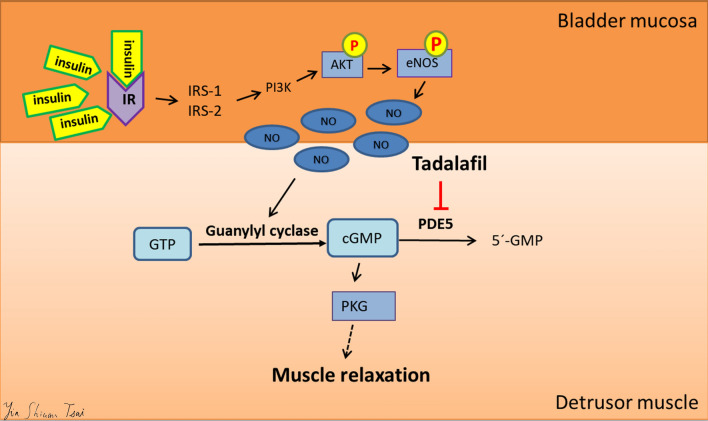
Schematic diagram illustrating the molecular events that take place in the bladder mucosa and detrusor, leading to the impairment of insulin-induced detrusor relaxation in metabolic syndrome (MS)-associated overactive bladder. High fructose diet induces hyperphosphorylation of IRS1 and IRS2, resulting in insulin resistance and the subsequent downregulation of the PI3K/Akt/eNOS signalling pathway and NO production in the mucosa. This is followed by the suppression of cGMP activity, and expression and level of PDE5 in the detrusor, leading to impairment of insulin-induced detrusor relaxation. Tadalafil treatment may ameliorate the MS-associated bladder overactivity through restoring insulin-activated detrusor relaxation via molecular repertories that are associated with increased eNOS phosphorylation in the bladder mucosa, restoration of urinary NO availability and detrusor cGMP level.

Results of the present study are interpreted to suggest that tadalafil may ameliorate MS-associated bladder overactivity by restoring insulin-activated detrusor relaxation through molecular mechanisms that are associated with preservation of the bladder mucosal IR/IRS/PI3K/AKT/eNOS pathway and detrusor cGMP production (Fig. 4). This study has several strengths. First, we provide new evidence for the increased serine phosphorylation levels of IRS1 (Ser^307^) and IRS2 (Ser^731^) as molecular mechanism of insulin resistance observed in the bladder mucosa of FFRs. Second, we demonstrated an alternative protective action of tadalafil, in addition to relaxing the urethral smooth muscle^11^, in treating OAB symptoms by improving insulin-activated detrusor relaxation. Third, we report new findings indicating different molecular mechanisms underlying the protective action of tadalafil versus metformin on OAB symptoms. Together, the current study highlights the significance of defected mucosal insulin signalling in MS-associated OAB; at the same time, suggests the mucosal IR/IRS/PI3K/AKT/eNOS pathway and detrusor cGMP production in the therapeutic action of metformin and tadalafil for the treatment of OAB under the condition of MS-associated insulin resistance.

This study supported the hypothesis that MS reduces insulin action in the bladder mucosa^7^; thus, impairs detrusor relaxation and contributes to bladder overactivity. Clinically, patients with insulin resistance developed OAB symptoms without overt neuropathy^19,20^. Insulin resistance is a key metabolic trait identified in rodent models of MS induced by dietary manipulation or genetic modification^15^. Insulin resistance can occur at multiple levels in cells of different tissues among various animal models^18^. For instance, Leiria et al.^7^ reported that AKT inactivation by endoplasmic reticulum stress contributes to the defected insulin action in the bladder mucosa, which results in bladder overactivity in obese mice. In the current study, we found that increased phosphorylation levels of IRS1 (Ser^307^) and IRS2 (Ser^731^) played a pivotal role in the negative regulation of insulin signalling in the bladder mucosa of FFRs, manifested by suppression of the downstream effectors (i.e., PI3K, AKT, and eNOS) and reductions in NOx and cGMP levels. Serine phosphorylation is believed to be deleterious to IRS signalling, particularly phosphorylation Ser^307^ in IRS-1^18^. In addition, IRS2 is a key active component in the action of insulin in tissues, and phosphorylated IRS2 (Ser^731^) is considered to be a counter regulator to the action of IRS2 and is involved in IRS2 disruption-mediated impaired insulin signalling^18,21^. It is noteworthy that metformin-treated FFRs restored the regulatory function of serine phosphorylation of IRS1 and IRS2 in the bladder mucosa and showed improvements on detrusor relaxation and bladder overactivity; whereas tadalafil exerted similar improvements on bladder overactivity in FFRs but no apparent effect on the increased phosphorylation levels of IRS1 (Ser^307^) and IRS2 (Ser^731^) in bladder mucosa. These data suggest metformin and tadalafil might target at different bladder tissues for their therapeutic effects on OAB. Metformin, as an insulin sensitizer, restores regulatory function of serine phosphorylation of IRS1 and IRS2 in the bladder mucosa; whereas tadalafil, as a PDE5 inhibitor, maintains the cGMP levels in the bladder detrusor.

Both NOS expression in and integrity of the bladder mucosa are prerequisite for the improvement on the insulin-dependent detrusor relaxation by tadalafil in FFRs. We found in the present study that the restoration of reduced detrusor relaxation to insulin stimulation in FFRs by tadalafil was significantly inhibited in the mucosa-intact bladder strips subjected to L-NAME treatment or in mucosa-denuded bladder. In addition, we showed that the daily oral intake of a low dose tadalafil for 4 weeks promoted eNOS phosphorylation at Ser^1175^ in the bladder mucosa of FFRs and restored both basal and insulin-stimulated increase in urinary NOx levels. Studies have reported that continuous treatment with PDE5 inhibitors, such as tadalafil and sildenafil, enhances eNOS activation and NOx levels in the heart and penis tissues^22,23^. Our findings are in accordance with those previous studies and suggest long-term intake of PDE5 inhibitors can benefit NO/cGMP production in the bladder tissue of FFRs. Another important factor in the control of cGMP levels in bladder tissues is the multi-drug resistance proteins (MRPs), an efflux pump for urinary cAMP and cGMP^24^. As such, the role of MRPs in restoration of cGMP level in the bladder detrusor of FFRs by tadalafil treatment warrants further investigation. In addition, increase in cGMP level has been reported to cause a feedback regulation of eNOS expression in cardiovascular system^25^. However, such a feedback regulation by cGMP on eNOS expression under tadalafil treatment was not observed in the present study. The existence of a feedback loop between the cGMP and NOS signalling in urinary bladder has not been identified^26^. Its clinical relevance also remains obscure.

The present study promotes the understanding the effects of dysregulated eNOS in the bladder mucosa on bladder overactivity in rats secondary to insulin resistance. In this study, we found the urinary NOx levels of the rats responded to the intraperitoneal injection of insulin in vivo, and the treatment with L-NAME inhibited the insulin-activated detrusor relaxation of rats in vitro. Furthermore, the expression levels of phosphorylated eNOS in the bladder mucosa were associated with the urinary NOx levels as well as subsequent cGMP production in the detrusor muscle in rats. The dominant isoform of NOS is eNOS, which is constitutively expressed in the mucosa^6,27^. The NO formed from urothelial eNOS was thought to inhibit bladder contraction, but the actual mechanism of action remains unclear. Our results provide a proof of concept to further elucidate the mechanism underlying the effect of mucosal eNOS on detrusor relaxation. Our findings also suggest that reduced insulin response in the bladder mucosa might have a role in the development of OAB symptoms through eNOS/NO/cGMP signalling.

It have been demonstrated that PDE5 inhibitors may exert their effect in muscle cells, nerve fibres, and interstitial cells of the bladder^28^. Given that bladder detrusor has the highest expression of PDE5 in lower urinary tract^29^, this prompted us to evaluate cGMP and PDE5 in detrusor muscle samples. It is intriguing to note that both the fructose and the fructose/tadalafil groups had a lower PDE5A2 activity in the bladder detrusor than the control group. PDE5 activity is one of the main mechanisms to regulate endogenous cGMP concentration; therefore, it is anticipated that as PDE5 is inhibited and cGMP levels restored by tadalafil treatment the expression/activity of PDE5 would be restored to control levels. Given that tadalafil binds on the active site of PDE5A to inhibit its enzymatic activity^30^ and the principle of ELISA for measurement of PDE5A activity is based on the production of 5′-GMP to the exogenous supply of its substrate cGMP in the kit, our findings could be interpreted to suggest that the activity of PDE5A remained to be suppressed due to the presence of tadalafil in the bladder lysates even the expression of PDE5A protein and tissue level of cGMP were restored in the tadalafil-treated group. A similar result was reported in another report^31^.

## Conclusions

A high-fructose diet may impair insulin signalling in the bladder mucosa. A high-fructose diet is associated with a decrease in urinary NOx and detrusor cGMP levels in rats, which can lead to the inhibition of detrusor relaxation and bladder overactivity. Our results indicate that daily tadalafil intake may ameliorate the MS-associated bladder overactivity through restoring insulin-activated detrusor relaxation via molecular repertories that are associated with increased eNOS phosphorylation at Ser^1175^ in the bladder mucosa, restoration of urinary NO availability and detrusor cGMP level.

## Material and methods

### Animals

This study was conducted in accordance with the guidelines of National Research Council, USA. The experimental protocol was approved by the Institutional Animal Care and Use Committee (Permit Number: 2015122802) of Kaohsiung Chang Gung Memorial Hospital and was carried out in compliance with the ARRIVE guidelines. All surgery was performed under urethane (1.2 g/kg) anaesthesia, and every effort was made to minimise the suffering of the animals and the number of animals used in our experiments.

Eighty female Wistar rats (BioLASCO Taiwan Co., Ltd., Taipei, Taiwan; weight: 200–240 g) were randomly allocated to 4 groups (n = 20) and subjected to an experimental course of 12 weeks. They were maintained in a facility accredited by the Association for Assessment and Accreditation of Laboratory Animal Care International under controlled temperature (24 °C ± 0.5 °C) and a light–dark cycle of 12 h each. For the experiments, rats were divided into 4 groups, namely control (regular chow), fructose (fructose group; fructose-rich diet; 60% fructose diet, Harlan Teklad, Madison Wisconsin), fructose plus tadalafil (tadalafil group; tadalafil 2 mg/kg, fed by gavage for 4 weeks beginning at age of 9 week), and fructose plus metformin (metformin group; metformin 20 mg/kg, fed by gavage for 4 weeks beginning at age of 9 week). Metformin, a widely-used biguanide anti-diabetic medication^32^, was included in the study as the treatment control of MS.

### Metabolic cage study, oral glucose tolerance test, and urinary NOx measurement

At the end of week 11, 12 rats from each group were placed in individual 3701M081 metabolic cages (Tecniplast, Buguggiate, Italy) equipped with an FT-104 force transducer (iWorx/CB Sciences, Inc., Dover, NH, USA), as reported previously^33^. After a familiarisation period (24 h), the volume of liquid consumed, micturition frequency, and urine output were measured for 3 days, and an average value was determined. Subsequently, the OGTT was performed after an overnight fast^33^. On the next day, we collected the urine of the rats before and 2 h after subcutaneous insulin injection (1 U/kg). NO oxidation was measured by the use of a NOx Colorimetric Assay kit (Cayman). The level of urinary NOx was considered as an estimate of bladder NO availability.

### Filling cystometry

Micturition cycles and voiding pressure were recorded according to the previously reported procedures^6,33^. In brief, twelve rats in each group were weighed and anaesthetised by subcutaneous injection of urethane (1.2 g/kg), followed by cannulation with a polyethylene-50 catheter into the left carotid artery to measure arterial pressure using a PowerLab 16S system with a P23 1D transducer (Gould-Statham, Oxnard, CA, USA). Through the urethra, another catheter was inserted into the bladder. The outlet of the catheter was connected to a T-tube, via which to a pressure transducer and a microinjection pump (CH-4103; Infors, Bottmingen, Switzerland). Room-temperature saline was infused into the bladder at a rate of 0.08 mL/min. Non-voiding contraction is defined as occurrence of a waveform on the filling phase of cystometry without the obvious interference of abdominal pressure; whereas voiding pressure is generated by an adequate continuous detrusor contraction that leads to bladder emptying. Cystometry was recorded using an RS3400 chart recorder (Gould, Cleveland, OH, USA). The animals were allowed to rest for a minimum of 30 min to ensure that the voiding pattern was stable. Reproducible micturition cycles were recorded for 1 h. Animals were sacrificed in the end of experiments by an overdose of urethane. Blood samples were collected for biochemical analysis.

### In vitro detrusor relaxation responses to insulin

Eight rats from each of the 4 groups were used. Full-thickness longitudinal strips with urothelium (10 × 2 mm) were prepared from the dorsal part of the bladder body. Each strip was connected to a force displacement transducer and was placed in a Compact Organ bath system (Panlab, SLU, Barcelona, Spain), and each chamber of the bath system contained 10 mL of physiological saline solution. The composition of physiological saline solution was 130 mM NaCl, 4.7 mM KCl, 1.17 mM MgSO_4_(H2O)_7_, 14.9 mMNaHCO_3_, 5.5 mM glucose, 1.6 mM CaCl_2_(H_2_O), 1.18 mM KH_2_PO_4_ and 0.03 mM CaNa_2_-ethylenediaminetetraacetic acid^34^. The solution was aerated using 95% O_2_–5% CO_2_ and maintained at 37 °C (pH 7.4). Resting tension in the tissues was controlled and fixed at 1 g. To facilitate the relaxation measurements during insulin stimulation, the bladder strips were soaked in organ bath containing KCl (80 mM) to induce a moderate contraction of strips. Then, cumulative concentration–response curves to insulin (10^−9^ to 10^−7^ M) were recorded in these pre-contracted bladder strips^7^. The insulin at the concentration of − 9 to − 7 M is a regular dosage in eliciting eNOS to produce NO in vitro studies^35^. Using the same procedure, the NO synthase inhibitor, nitro-l-arginine methyl ester (L-NAME) (100 μM), was added to some preparations to inhibit NO production. In a separate series of experiments, the insulin-induced detrusor relaxation was conducted in mucosa-denuded bladder strips (n = 6, in each of the 4 groups), of which the mucosal layer was scraped off carefully under a dissecting microscope before mounting in the organ bath. Contraction responses were recorded as tension (g) per cross-sectional area (mm^2^), and relaxation responses were represented as the percentage of the amplitude of the KCl-induced pre-contraction.

### Western blots of bladder mucosa and detrusor proteins and measurement of PDE5 activity and cGMP levels for the detrusor

Naïve bladder bodies were divided into mucosa and detrusor layers by microdissection^6,16^. Western immunoblotting was performed using the bladder mucosa layer for the canonical signalling pathway of insulin and the detrusor layer for PDE5 expression, as previously described^33^. In brief, alternative samples from each group were homogenised on ice in CelLytic^TM^MT cell lysis buffer (Sigma-Aldrich) containing a protease inhibitor. The total protein was measured using the Pierce 660-nm protein assay (Thermo, Waltham, MA, USA). Sodium dodecyl sulphate–polyacrylamide gel electrophoresis was performed using the Laemmli buffer system.

Antibodies raised against insulin receptor (IR) (1:200 dilution; Santa Cruz), phospho-IR (Tyr1150) (1:200 dilution; Santa Cruz), insulin receptor substrate (IRS) 1 (1:200 dilution; Santa Cruz), phospho-IRS1 (Ser307) (1:500 dilution; Millipore), IRS2 (1:1000;ABclona), phospho-IRS2 (Ser731) (1: 1000 dilution; Abcam), PI3K (1:1000 dilution; Abcam), phospho-PI3Kp85 (Tyr508) (1:500 dilution, Santa Cruz), AKT (1:1000 dilution; Cell Signal), phospho-AKT (Ser473) (1:1000 dilution; Cell Signal), eNOS (1:1000 dilution; Cell Signal), phospho-eNOS(Ser1175) (1:200 dilution; Santa Cruz), PDE5A (1:500 dilution; Santa Cruz), and glyceraldehyde 3-phosphate dehydrogenase (GAPDH) (1:10,000 dilution; Millipore) were used. The validation of antibodies used in this study is listed in Supplementary Table S1.

Detrusor muscle samples from the rats were used for measuring PDE5 activity and cGMP levels by a PDE5A2 activity ELISA kit (MyBioSource, CA, USA) and a cGMP ELISA kit (Ann Arbor, MI, USA), according to the manufacturer’s protocol. The assays were performed in duplicates, and the sample weight was used to calibrate the data.

### Statistical analysis

All data are presented as the mean ± standard error of mean (SEM). Data were subjected to one-way analysis of variance and multiple comparisons by using the Dunnett test. Paired *t* testing was also used. For all statistical tests, *P* < 0.05 was considered statistically significant.

## Supplementary Information

Supplementary Information.
