# Supplementary material for: Tadalafil ameliorates bladder overactivity by restoring insulin-activated detrusor relaxation via the bladder mucosal IRS/PI3K/AKT/eNOS pathway in fructose-fed rats
Source: Sci Rep. 2021 Apr 15;11:8202. doi: 10.1038/s41598-021-87505-3 (PMC8050041; doi:10.1038/s41598-021-87505-3)
Supplement: Supplementary file 1 — Supplementary Information. [file 41598_2021_87505_MOESM1_ESM.pdf]

Tadalafil ameliorates bladder overactivity by restoring insulin-activated detrusor relaxation via the bladder mucosal IRS/PI3K/AKT/eNOS pathway in fructose-fed rats

Wei-Chia Lee, M.D., Ph.D.[1], Steve Leu, Ph.D. [2], Kay L.H. Wu, Ph.D.[2], You-Lin Tain, M.D., Ph.D. [3], Yao-Chi Chuang M.D.[1], Julie Y. H. Chan* Ph.D. [2]

[1]Division of Urology, Kaohsiung Chang Gung Memorial Hospital, and Chang Gung University College of Medicine, Kaohsiung, Taiwan

[2]Institute for Translational Research in Biomedicine, Kaohsiung Chang Gung Memorial Hospital, Kaohsiung, Taiwan

[3]Department of Pediatrics, Kaohsiung Chang Gung Memorial Hospital, and Chang Gung University College of Medicine, Kaohsiung, Taiwan.

Running title: Insulin signalling in amelioration of bladder overactivity by tadalafil.

***Address correspondence and reprint requests to**: Julie Y. H. Chan Ph.D.

Institute for Translational Research in Biomedicine, Kaohsiung Chang Gung Memorial Hospital, 123 Ta Pei Rd., Niao Song Qu, Kaohsiung City, Taiwan, Republic of China, e-mail: jchan@cgmh.org.tw, Tel. +886-77338415, Fax: +886-77338415.

Supplementary Table S1

The information of antibodies used in the present study.

Antibody	catalog	Reference
Insulin Rβ (46) IR	SC-135949	Shao, X. *et al*. Sci Rep **6,** 35482 (2016).
p-insulin Rβ Antibody (10C3)p-IR	SC-81500	Hua, Y. *et al.* Mol Med Rep **15,** 180-186 (2016).
IRS-1 Antibody (E-12)	sc-8038	Jelena, S. *et al.* Mol Cell Endocrinol **420,** 97-104 (2015).
Anti-phospho-IRS1 Antibody (Ser302mouse/ Ser307human)	05-1086	Hancer, N. J. *et al.* J Biol Chem **289,** 12467-12484 (2014).
IRS2 Rabbit pAb	A7945	Hsu, M. H. *et al.* Int J Mol Sci **21,** 3428 (2020).
Anti-IRS2 antibody (phospho S731)	ab3690	Sun, Z. *et al.* BMC Complement Altern Med **16,** 200 (2016).
Anti-PI3 Kinase p85 α antibody	ab191606	Luan, Y. *et al.* Front Cell Neurosci **11,** 285 (2017).
PI 3-kinase p85α (Tyr 508)	sc-24619	Manabu, U. *et al.* Biofactors **39,** 457-66 (2013).
Akt (pan) (C67E7) Rabbit mAb	#4691	Huang, D. *et al.* Sci Rep **6,** 26059 (2016).
Phospho-Akt (Ser473) Antibody	#9271	Yang, H. *et al.* Mol Med Rep **13,** 49-58 (2016).
eNOS (D9A5L) Rabbit mAb	#32027	Huisheng, W. *et al.* Exp Ther Med **16,** 1195-1202 (2018).
p-NOS3 Antibody (15E2)	sc-81510	Tovar, A. R. *et al.* J Nutr **138,** 462-468 (2008).
PDE5A Antibody (D-3)	sc-398747	West, T. M., *et al.* J Am Heart Assoc **8,** e012273 (2019).

Supplementary Figure S2

The entire unmodified gels/images illustrated in figure 3.

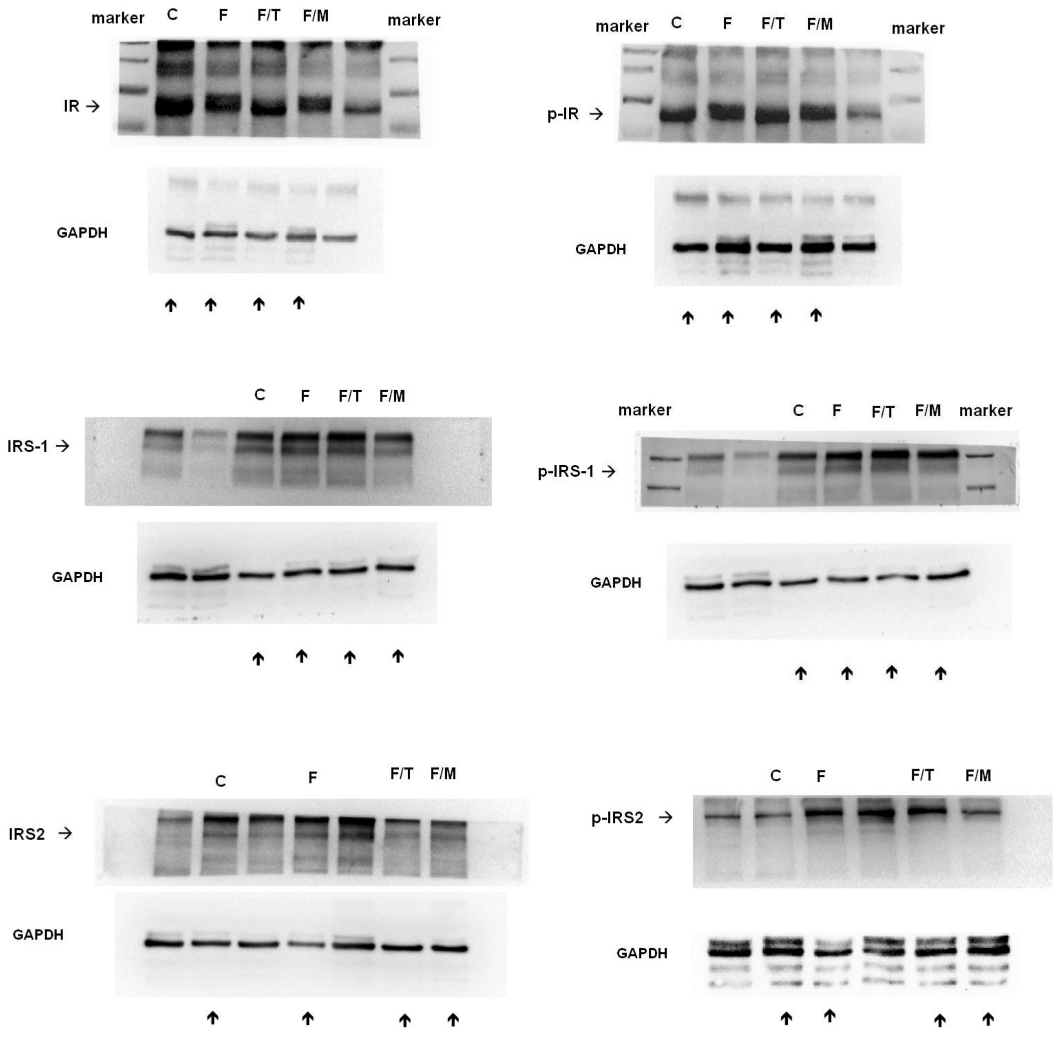

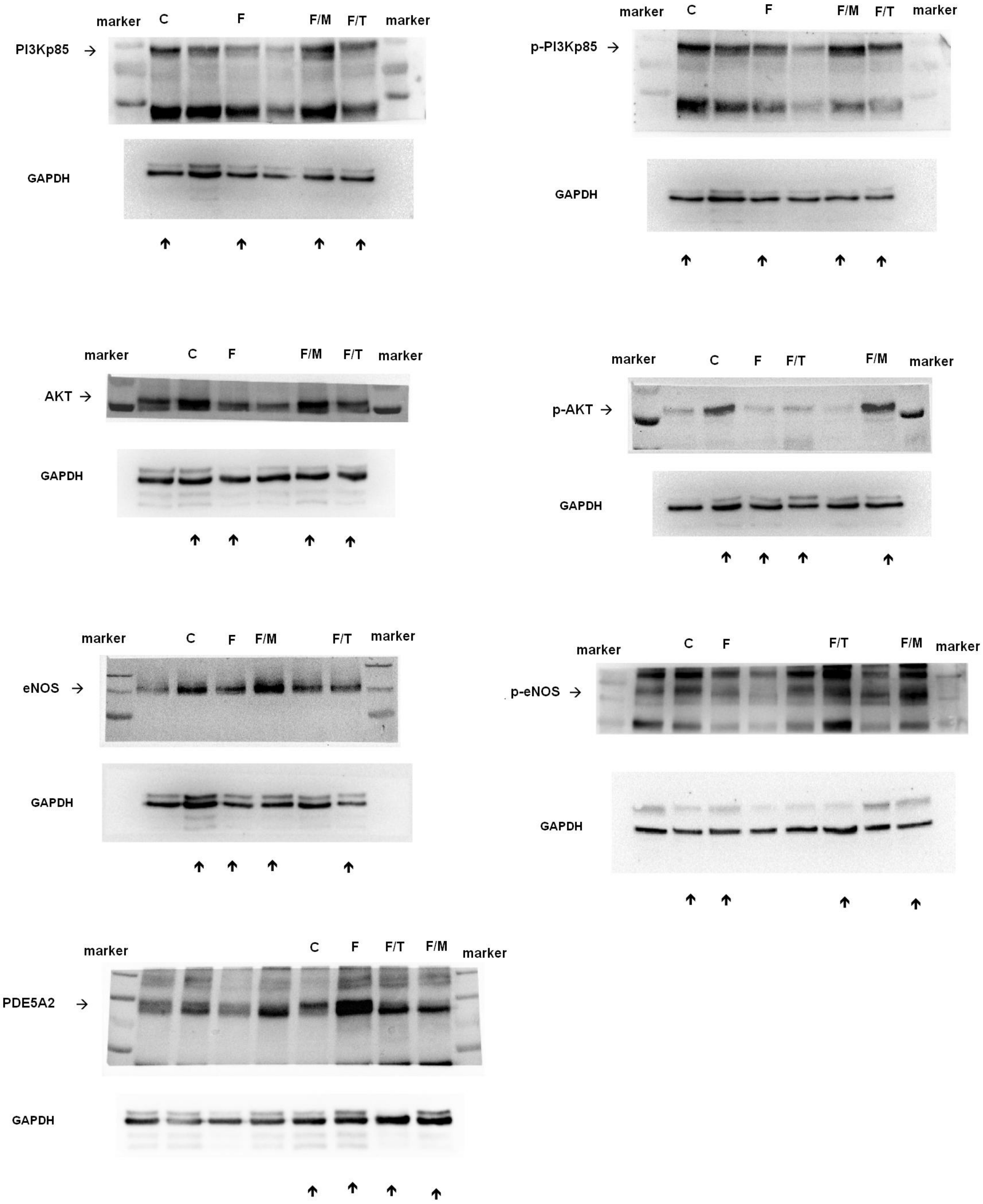

marker
C
F
F/M
F/T
marker
PI3Kp85
GAPDH
marker
C
F
F/M
F/T
marker
p-PI3Kp85
GAPDH
marker
C
F
F/M
F/T
marker
AKT
GAPDH
marker
C
F
F/T
F/M
marker
p-AKT
GAPDH
marker
C
F
F/M
F/T
marker
eNOS
GAPDH
marker
C
F
F/T
F/M
marker
p-eNOS
GAPDH
marker
C
F
F/T
F/M
marker
PDE5A2
GAPDH
